# Compact Bandwidth-Enhanced 180-Degree Phase Shifter Using Edge-Coupled Multi-Microstrip and Artificial Transmission Line

**DOI:** 10.3390/mi14091692

**Published:** 2023-08-29

**Authors:** Ding He, Jingxin Fan, Zhiqiang Zhu, Yang Yuan, Zhongjun Yu

**Affiliations:** 1 Aerospace Information Research Institute, Chinese Academy of Sciences, Beijing 100190, China; heding201@mails.ucas.ac.cn (D.H.); fanjingxin21@mails.ucas.ac.cn (J.F.); yuanyang19@mails.ucas.ac.cn (Y.Y.); 2 School of Electronic, Electrical and Communication Engineering, University of Chinese Academy of Sciences, Beijing 101408, China; zzq_1633@163.com; 3 Institute of Information Engineering, Chinese Academy of Sciences, Beijing 100085, China

**Keywords:** artificial transmission line, bandwidth enhancement, compact, edge coupled, GaAs, microstrip line, phase shifter

## Abstract

Compactness has obtained sufficient importance in wideband phase shifter design considerations, as it is directly related to fabrication cost. In this paper, a novel structure was presented to create compact broadband 180-degree phase shifter, which has the advantages of enhanced bandwidth and significantly reduced chip area. The proposed configuration consists of edge-coupled multi-microstrip lines (ECMML) and an artificial transmission line (ATL) with dual-shorted inductors, both of which have the periodic shunt load of capacitors. The ECMML can provide a high coupling coefficient, leading to an increase in the bandwidth, while the introduced capacitors can greatly reduce the line length (35.8% of the conventional method). To verify the relevant mechanisms, a wideband switched network with compact dimensions of 0.67 × 0.46 mm^2^ was designed via 0.15-micrometer GaAs pHEMT technology. Combined with the measured switch transistor, it was shown that the proposed phase shifter exhibits an insertion loss of less than 2 dB, a return loss of greater than 12 dB, a maximum phase error of less than 0.6° and a channel amplitude difference of less than 0.1 dB in the range of 10 to 20 GHz.

## 1. Introduction

With the emergence of the Internet of Things (IoT) and fifth-generation (5G) communication, demand for technological innovation has also driven modern wireless systems, transceivers, and standards to evolve with rapid speed [1,2]. As one of the most crucial components in these radio frequency (RF) systems, along with modulators, multiple-input multiple-output (MIMO) or phased arrays, phase shifters are primarily employed to generate relatively constant phase difference for the achievement of electronic beam forming and scanning [3,4,5,6]. Therefore, its performance directly affects the essential metrics of the system. Typically, phase shifters can be classified as digital, analog and mixed digital–analog types [7]. Compared to analog methods that use continuous phase variation, the mainstream solutions prefer a digital-based approach, since it allows more precise phase control at pre-determined states through switching [8,9].

With regard to broadband phase shifter design, compactness has become one of the foremost considerations, which is mainly due to the following reasons: (1) In recent years, the trend of highly integrated electronic devices has led to severe accentuation of limited space and required a tighter distribution of chip layouts, which is strongly correlated with low manufacturing costs [10]. (2) In addition, long signal lines normally produce a high level of insertion loss, which can significantly reduce the signal-to-noise ratio (SNR) of the receiver and deteriorate the output power of the transmitter. Therefore, with these situations in mind, phase shifters with a compact size, low insertion loss, good return loss and minimized phase variation over the broadband range have received extensive attention.

Currently, in order to build the abovementioned broadband phase shifter, numerous studies have concentrated on transmission-type circuits [11,12,13,14,15,16,17,18,19,20], in which the passive phase-shifting part is composed of lumped components or distribution networks. Among these techniques, the switched-line type is the simplest method, as it achieves a phase difference proportional to frequency through switching transmission paths of different electrical lengths [11,12]. However, this method exhibits a narrow band and performs high insertion loss behavior due to the long lines utilized. The classical and modified Schiffman phase shifter [13,14,15,16,17] is well known for its broadband characteristics, and it uses a uniform transmission line as its reference and a coupled section as its main line to obtain the desired phase shift. Nevertheless, due to the limited coupling coefficient, it is extremely difficult to use it in applications beyond 90°. Equally, the high/low pass network type [18] and broadside-coupled structure [19] also suffer from poor bandwidth performance at large phase shifts. Fortunately, it is worth noting that the method proposed in [20] using parallel-coupled lines can theoretically achieve the ideal 180° phase difference at any frequency band. Moreover, it is particularly suitable for implementation in GaAs monolithic microwave integrated circuits (MMIC), in which only two metal layers exist and, thus, must be designed in edge-coupled form. However, the drawback of using this approach is that it still requires a quarter waveguide wavelength line, resulting in a large area consumption.

To address the above issues, this paper presents a novel switched network based on a reverse short-circuited coupled line for a broadband 180-degree phase shifter design. The proposed circuit configuration comprises edge-coupled multi-microstrip lines (ECMML) as Path 1 and an artificial transmission line (ATL) as Path 2, which is symmetrically loaded on both sides with dual-shorted inductors. In contrast to conventional phase shifters, the proposed phase shifter has the following advantages: (1) In Path 1, compared to traditional dual-coupled line structure, the desired tight-coupling performance can be obtained by adjusting the parameters of the proposed ECMML to significantly enhance the bandwidth. (2) In Path 2, grounded inductors are utilized instead of quarter waveguide wavelength shorted stubs, which not only reduce the area, but also allow more flexibility in balancing the amplitude error between Path 1 and Path 2. (3) Different values of shunt capacitors are employed in each of the two paths, which can greatly decrease the physical lengths of the phase shifter and contribute to its applications in compact situations. In addition, the specific theoretical analysis and derivation of formulas are given for more detailed explanations. Based on the proposed technique, a novel 180-degree phase shifter is designed via 0.15-micrometer GaAs pHEMT technology to perform demonstration, which has excellent performance in terms of compact size compared to other wideband phase shifters.

## 2. Theoretical Analysis of the Reverse Shorted Coupled Line Prototype

**Figure 1:**
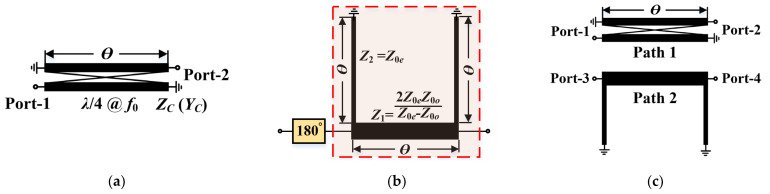
(**a**) Reverse shorted coupled line; (**b**) Equivalent circuit; (**c**) Phase shifter prototype.

In order to better explain the principle of the proposed 180° phase shifter, we started from the analysis of the reverse coupled line. Its schematic and equivalent circuit are [21,22] shown in Figure 1a,b, where *θ* represents the electric length of the transmission line, *Z_C_* represents the characteristic impedance of the coupled line and is equal to [*Z*_0*e*_ *× Z*_0*o*_]^1/2^, *Z*_1_ (*Y*_1_) represents the characteristic impedance (admittance) of the main transmission line in Figure 1b, and *Z*_0*e*_ (*Y*_0*e*_) and *Z*_0*o*_ (*Y*_0*o*_) represent the even- and odd-mode impedances (admittances) of the coupled line, respectively.

(1) Impedance matching analysis and drawback-1: When Port-2 is terminated with *Z_C_*, the input admittance of Port-1 (i.e., *Y_in_*) is

(1) $$Y_{in}=\frac{1}{Z_{in}}=Y_{1}\frac{Y_{F}+jY_{1}\tan\theta }{Y_{1}+jY_{F}\tan\theta }-jY_{0e}\cot\theta ,$$

where *Y_F_* = *Y*_0_ − *jY*_0*e*_cot*θ* (this variable is defined only for ease of writing), and *Y*_1_ = (*Y*_0*o*_ − *Y*_0*e*_)/2. In order to maintain a good match at Port-1, it is necessary to make the input impedance *Z_in_* = *Z_C_*. Substituting this into Equation (1) and solving for it yields

(2) $$Z_{in}=Z_{C}=\frac{2Z_{0e}Z_{0o}\sin\theta }{{\left[{\left(Z_{0e}-Z_{0o}\right)}^{2}-{\left(Z_{0e}+Z_{0o}\right)}^{2}\cos^{2}\theta \right]}^{\frac{1}{2}}}.$$

Note that the above equation indicates an important conclusion: for the coupled line in Figure 1a to maintain well-matched performance at Port-1, *Z_C_* must satisfy both *Z_C_* = [*Z*_0*e*_ × *Z*_0*o*_]^1/2^ and the calculated value of Equation (2). However, *Z_C_* is actually a frequency-independent constant. In other words, the coupled-line structure can only achieve the best match at a single frequency point, while a mismatch occurs at other frequency points. It is also worth mentioning that *Z_in_ = Z_c_ =* 2(*Z*_0*e*_ × *Z*_0*o*_)/(*Z*_0*e*_ − *Z*_0*o*_) = *Z*_1_ is valid only when *θ* = 90°. At this time, the symmetrically shunted shorted stubs can also be regarded as an open circuit, resulting in a good match. That is to say, the conventional coupled-line structure has an obvious drawback: its physical length must be set to one-fourth of the center-frequency wavelength, which seriously restricts its application in small-size situations.

(2) Amplitude/phase characterization and drawback-2: The amplitude/phase characteristics of Figure 1a are summarized in [20,21,22], where it is deduced that the signal–voltage ratio between Port-1 and Port-2 is constant at 1. Obviously, this illustrates that this reverse coupled-line structure has the frequency-independent property and can transmit signals with equal amplitude if line losses are not taken into account. Furthermore, the phase difference *β* between Port-1 and Port-2 can be derived as

(3) $$\beta =\cos^{-1}\left[\left(\frac{Z_{0e}+Z_{0o}}{Z_{0e}-Z_{0o}}\right)\cos\theta \right].$$

When *Z*_0*e*_ is much larger than *Z*_0*o*_, *β* is approximately equal to *θ*. Thus, the total phase shift of the reverse coupled line is 180° + *β =* 180° + *θ*. In other words, if Path 2 in the phase shifter takes the form of the transmission line circled by the red dashed line in Figure 1b, it can theoretically produce a phase difference of 180° + *β* − *θ* = 180°. Note that this conclusion only holds if the coupling coefficient is close to 1, which is virtually impossible to achieve. In summary, although the phase shifter in Figure 1c can theoretically achieve a 180° phase shift with equal amplitude over the full frequency band, it requires that the coupled line must have a sufficient coupling coefficient *C*, which is a challenge for a dual-coupled line and hence a major limiting factor in its performance.

**Figure 2:**
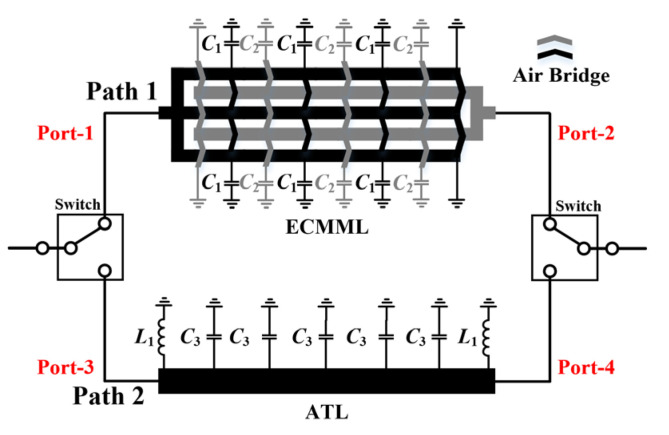
Schematic of the proposed phase shifter.

Aiming at the drawbacks of the traditional dual-coupled line phase shifters, this paper proposes an improved broadband compact phase shifter consisting of ECMML and ATL, whose schematic is shown in Figure 2. ECMML can effectively improve the coupling coefficient, which is beneficial to the enhancement of phase difference performance and bandwidth. Moreover, the ATL can accurately compensate for the phase while significantly reducing the chip size, thus further improving the phase difference close to the desired 180°. The specific principles of the proposed techniques are explained in detail below.

## 3. Analysis of the Proposed Technologies in Novel 180° Phase Shifter

### 3.1. Effects and Selection of ECMML

**Figure 3:**
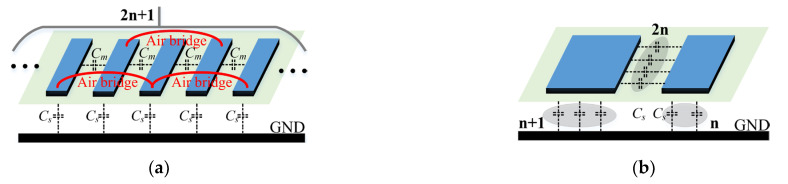
Schematics of (**a**) Asymmetric multi-microstrip edge-coupled line model; (**b**) Simplified model of ECMML.

**Figure 4:**
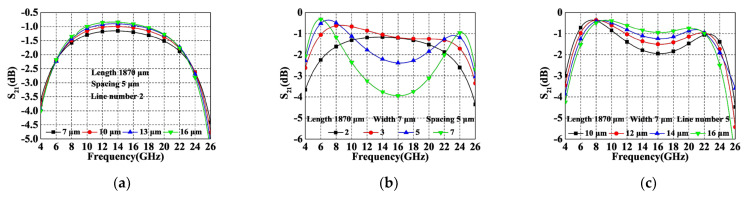
Simulated IL of ECMML with various parameters. (**a**) Width; (**b**) Number; (**c**) Spacing.

From the discussion in Section 2, it is clear that the two coupled lines in Figure 1c only provide a limited coupling coefficient, which is detrimental to the phase difference. Hence, an asymmetric multiconductor edge-coupled line model is proposed to improve *C* as shown in Figure 3a, where *Cs* represents the capacitance between the conductor and ground, and *Cm* refers to the mutual capacitance between adjacent lines. For clarity of analysis, it can be equated to the simplified asymmetric parallel coupled line in Figure 3b. Hence, the improved coupling coefficient *C* is given by

(4) $$C=\sqrt{\frac{4n^{2}C_{m}^{2}}{\left[\left(n+1\right)C_{s}+2nC_{m}\right]\left(nC_{s}+2nC_{m}\right)}}=\sqrt{\frac{1}{\left(0.25+0.25/n\right)k^{2}+\left(1+0.5/n\right)k+1}},$$

where 2*n* + 1 stands for the total number of microstrip lines and *k* represents a capacitance ratio of *C_s_*/*C_m_*. However, it must be noted that although a larger *C* favors enhanced phase difference and bandwidth, the optimal coupling coefficient is not designed to be at its highest point, since it is also limited by in-band insertion loss requirements (over-tight coupling increases the insertion loss within 10–20 GHz). Therefore, the coupling coefficient should be a trade-off between bandwidth and insertion loss. Based on (4), there are three factors affecting *C*, i.e., *C_s_*, *C_m_*, and *n*. The corresponding design parameters are line width *W*_1_, line spacing *d*_1_ and a number of coupled lines. For visualization purposes, Table 1 summarizes the effect of these parameters on the coupling coefficient. A simulation is subsequently provided in Figure 4 to verify its effectiveness (the line length is fixed at 1870 µm, which is a quarter-wavelength line at 15 GHz). As immediately seen, the bandwidth increases as *W*_1_ decreases, *d*_1_ decreases and 2*n* + 1 increases. In addition, it can be observed from Figure 4b that when the number of lines is 2 and 3, the bandwidth and low in-band insertion loss cannot be achieved simultaneously even at the minimum spacing and line width, so the number of parallel microstrip line should be chosen to be greater than 3. On the basis of balancing the insertion loss and the bandwidth, the final parameters of the ECMML were selected as 7 µm *W*_1_, 14 µm *d*_1_, and line number of 5.

Note that the results in Figure 4b correspond to the maximum coupling coefficients for each number of lines, and only illustrates that a larger coupling coefficient and wider bandwidth can be obtained with 7 lines than with 5 lines. If 7 lines were chosen, the relatively favorable performance over 5 lines would have to be obtained by increasing the line width and line spacing, but this approach was not used due to the greater size of the area consumed.

### 3.2. Size Reduction in ATL

**Figure 5:**
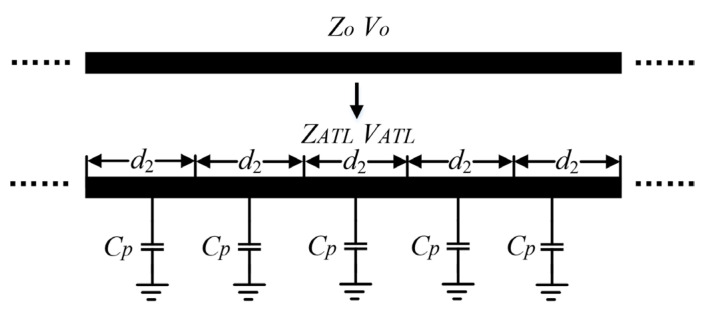
Schematic of the proposed ATL with periodic shunt capacitors.

According to the analysis in Section 2, another pressing disadvantage of the conventional dual-coupled line structure is that it cannot be directly applied in compact applications due to the presence of quarter-wavelength lines, which occupy a large circuit area, especially at low frequencies. Fortunately, researches [23,24,25] have shown that the ATL concept is applicable to reduce the physical size of planar circuits. Therefore, in order to adequately reduce the size of the proposed 180° phase shifter, we convert the transmission line in Path 2 of Figure 1c into ATL form, as shown in Figure 5. Since the lengths of Path 1 and Path 2 are identical, only Path 2 is shown here as an example.

As seen in Figure 5, when the periodic shunt capacitors are loaded equally spaced onto the transmission line with characteristic impedance of *Z*_0_ and phase velocity *v*_0_, the phase velocity of the main line decreases to *v_ATL_*, which can be deduced as

(5) $$v_{ATL}=\frac{1}{\sqrt{L_{0}\left(C_{0}+\frac{C_{p}}{d_{2}}\right)}},\;v_{0}=\frac{1}{\sqrt{L_{0}C_{0}}},$$

where *d*_2_ represents the periodic spacing, *C_p_* is the mounted ground capacitor, and *L*_0_ and *C*_0_ refer to the distributed inductance and capacitance of the main transmission line, respectively. Compared to *v*_0_, *v_ATL_* is significantly smaller, which means that a larger phase shift can be achieved with the same or smaller physical dimensions of the transmission line after the introduction of the shunt capacitors. In other words, Equation (5) reveals an important conclusion: when the total phase shift is fixed, the physical length of the ATL loaded with shunt capacitance is shorter than that of a normal microstrip line without shunt structure. For an *N*-section ATL, the total phase shift available $\phi_{ATL}$ can be calculated as

(6) $$\phi_{ATL}=\frac{Nd_{2}\omega_{0}}{v_{ATL}}=Nd_{2}\omega_{0}\sqrt{L_{0}\left(C_{0}+\frac{C_{p}}{d_{2}}\right)}$$

where *ω*_0_ is the center angular frequency of interest. According to Equation (6), to keep the total length *N* × *d*_2_ of ATL as small as possible, it is necessary to decrease *d*_2_ and increase the value of *C_p_* while increasing *N*. It is worth mentioning that due to process limitations, the ground pads behind the capacitors cannot be placed too close to each other. Hence, spacing *d*_2_ cannot be too small, which also prevents mutual coupling of the metal layers of adjacent capacitors. Similarly, the ECMML in Path 1 is also shortened by using shunt capacitors, which are evenly distributed on both sides of the ECMML to maintain the symmetry of the pattern as much as possible. Moreover, the air bridges used for spaced-line connections can also provide additional mutual capacitance *Cm* to improve coupling. After iterations of the above-mentioned approach, the size of the final phase shifter was significantly reduced from 1870 µm to 670 µm, which is only 35.8% of the conventional structure and validates the effectiveness of introducing the ATL technique.

In addition, to further save the area, the dual-shorted quarter-wavelength stubs in Path 2 were replaced by two spiral inductors. This had the additional benefit of greater inductor losses, which reduced the quality factor *Q*, expanded the bandwidth and simultaneously facilitated the balancing of the amplitude difference between Paths 1 and 2.

### 3.3. Precise Control of Phase Shift

**Figure 6:**
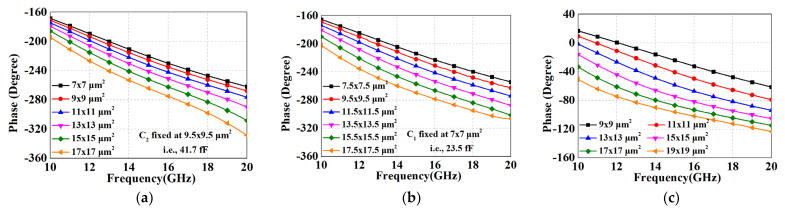
Simulated results. (**a**) Phase shift vs various *C*_1_ in Path 1; (**b**) Phase shift vs various *C*_2_ in Path 1; (**c**) Phase shift vs various *C*_3_ in Path 2.

Although the ECMML technique in Section 3.1 is effective in increasing the coupling coefficient, it is still not possible to achieve a coupling coefficient of 1. Moreover, considering the requirements of low insertion loss, the coupling coefficient is not set at its maximum value. This means that the desired 180° phase difference cannot be achieved by relying on the coupled line structure alone. Fortunately, it is known from the analysis in Section 3.2 that the shunt grounded capacitances allow for a greater phase shift of the transmission line. Hence, we apply these shunt capacitors, *C*_1_, *C*_2_ and *C*_3_, in ECMML and ATL to further control the phase shift. Figure 6 shows the phase shift simulation results for different capacitance values in each path. It can be noticed that the phase shifts in each path become larger as the capacitance values increase, as predicted by Equation (6). Based on the above monotonically controllable property, the phase difference of the phase shifter can be easily maintained around the 180° curve over a wide bandwidth by continuously iterating different combinations of shunt capacitors.

**Figure 7:**
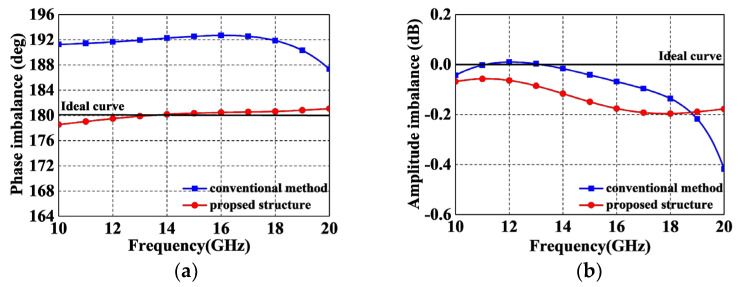
Performance comparisons. (**a**) Phase imbalance; (**b**) Amplitude imbalance.

Figure 7 demonstrates the comparison of the amplitude/phase performance between the conventional method and the proposed novel structure. From Figure 7a, it can be seen that the phase difference of the conventional method in Figure 1c is deteriorated due to the insufficient coupling coefficient, whereas the amplitude and phase difference performances of the proposed structure are both enhanced and close to the ideal curves. These strengths are exactly attributed to the high coupling coefficient of the ECMML and the fact that the phase difference can be further accurately compensated by the shunt capacitors.

## 4. Broadband 180° Phase Shifter Design and Its Performance

### 4.1. Simulated Performances of the Novel Switched-Network in Path 1 and Path 2

**Figure 8:**
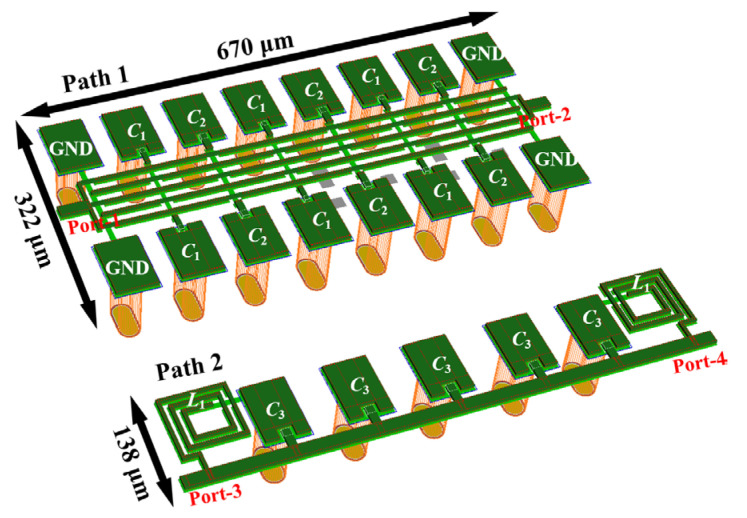
Layout of the proposed 180-degree phase shifter.

A novel broadband compact switched-network for the 10–20 GHz phase shifting was finally realized via 0.15 µm GaAs pHEMT technology, as shown in Figure 8. The dimensions of Path 1 are 670 × 322 µm^2^, with a line spacing of 14 µm, a line width of 7 µm, and load capacitances of 0.0235 pF and 0.0417 pF for *C*_1_ and *C*_2_, respectively. All the parameters are adjusted according to the electromagnetic (EM) simulation results. The dimensions of Path 2 are 670 × 138 µm^2^, with a periodic structure spacing of 120 µm, a main line width of 20 µm, and load capacitance *C*_3_ and inductance *L*_1_ of 0.055 pF and 0.56 nH, respectively.

**Figure 9:**
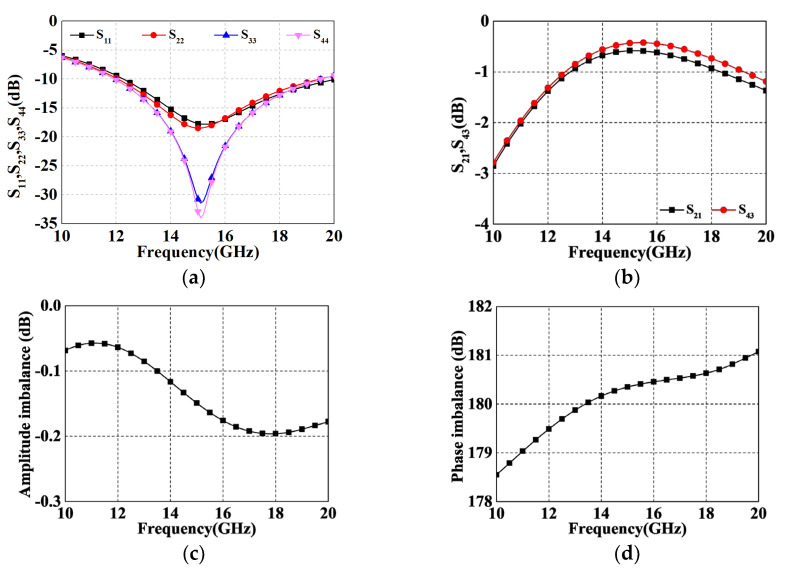
EM simulated results. (**a**) *S*_11_, *S*_22_, *S*_33_, and *S*_44_ (i.e., return loss of each port); (**b**) *S*_21_ and *S*_43_ (i.e., insertion losses for Path 1 and Path 2); (**c**) Amplitude difference; (**d**) Phase difference.

Figure 9 shows the corresponding EM simulation results for both paths, which are simulated without the switching tubes. Figure 9a shows the simulated return loss (RL) of each port with respect to the operating frequency when the termination impedance is fixed at 50 Ω. Both channels show an RL below −6 dB from 10 to 20 GHz with a slight deterioration at a low frequency band. Figure 9b illustrates the insertion loss of the design, i.e., *S*_21_ and *S*_43_. Profited from the prominent performance of ECMML and ATL, the minimum insertion loss (IL) of both paths is around 0.45 dB, and the average values are 1.6 dB and 1.71 dB, respectively. It is worth noting that the increase in the insertion loss at low frequency bands can be attributed to the deterioration of RL. Moreover, as shown in Figure 9c, the two IL curves are very tightly fitted together due to the proposed technique, resulting in a superior balanced performance in terms of amplitude difference (less than 0.2 dB over the whole operating frequency band). The same excellent antiphase characteristic can be observed in Figure 9d, where the phase error is kept within ±1.5°.

### 4.2. Performances of Complete 180° Phase Shifter with Measured Switching Transistors

**Figure 10:**
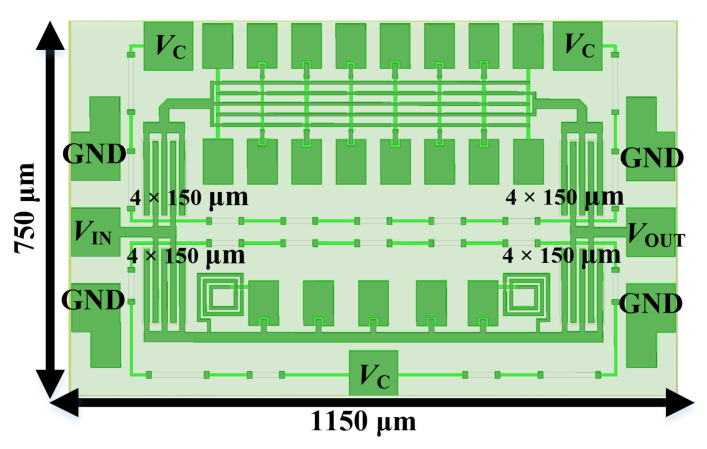
Overall view of the phase shifter with switch transistors.

In fact, phase shifters need to be used in conjunction with switches. Hence, in order to verify the effectiveness of the proposed novel switch-network in MMIC design, a complete 180-degree phase shifter module is specifically constructed using 4 × 150 µm transistors as switching tubes, as shown in Figure 10. By interlinking the source and drain, transistors are biased in diode-connected configurations, which are switched on/off by a control voltage *V*_c_ of 0/−5 V. In addition, the data for the different states are provided by an on-wafer measurement system based on a four-port vector network analyzer (Ceyear 3672E made by Ceyear, Qingdao, Shandong, China). It is important to note that since transistors are not ideal switches, the introduction of the transistor changes the impedance of the input/output ports (which is not equivalent to the on-path alone), and it is actually a parallel connection of two paths. Considering that the large size of the transistor is favorable to reduce the IL of the on-path, the final trade-off is to sacrifice some turn-off performance by sizing it at 4 × 150 µm.

**Figure 11:**
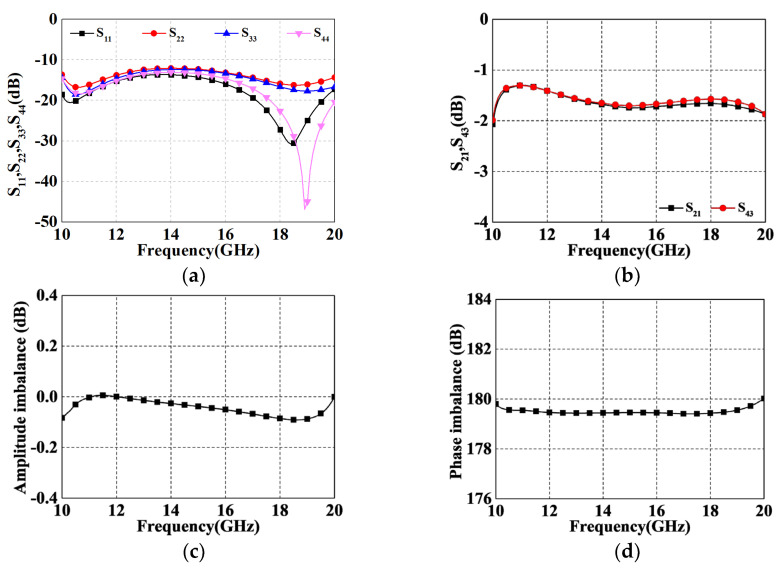
Performance with measured switching transistors. (**a**) *S*_11_, *S*_22_, *S*_33_, and *S*_44_ (i.e., return loss of each port); (**b**) *S*_21_ and *S*_43_ (i.e., insertion losses); (**c**) Amplitude difference; (**d**) Phase difference.

The corresponding performance of the complete design is shown in Figure 11, which is better characterized than the simulation results without transistors in Figure 9. This is because the impedance at the input/output ports is equal to the parallel connection of the on-path and the other off-path. Consequently, it optimizes the port matching by smoothing the in-band impedance to improve the performance. Figure 11a illustrates that from 10 to 20 GHz, all the optimized RL curves are below −12 dB. Clearly, there is a significant improvement in RL compared to the results in Figure 9a. It can also be observed in Figure 11b that IL is also improved due to the enhancement of the standing wave performance, and the loss in the low frequency band is reduced, with a smooth response in the range of −1.3 dB to −2 dB across the entire frequency band. In addition, the amplitude imbalance and the phase errors are also significantly refined due to the above-mentioned enhancements. Figure 11c shows that the amplitude difference between two IL curves almost coincides around 0 dB, with a maximum error of only 0.1 dB. Figure 11d shows that the phase difference is also maintained at 180° over a wide bandwidth, with a maximum error of 0.6°.

Table 2 summarizes the performance of the designed phase shifter and compares it with other reported state-of-the-art methods. According to the reference data, the proposed phase shifter with novel switch-network consumes less area and achieves more balanced amplitude and phase characteristics in the 10 to 20 GHz range. Note that the dimensions used in Table 2 are normalized by *λ_g_* in the corresponding frequency bands. Therefore, comparisons can be made even if the design bands are different.

## 5. Conclusions

In this paper, a novel broadband switch-network composed of ECMML and ATL is presented for compact passive 180° phase shifter. Through analyzing the reverse coupled line and designing the multi-parallel microstrip lines, the coupling coefficient can be improved, thus effectively extending the bandwidth. Due to the introduction of periodic load capacitance in ECMML and ATL, the proposed switch-network achieves a compact chip size, which provides the possibility of high integration of transmission line-based phase shifters. Compared with other works, the designed 180° phase shifter achieves a precise phase difference with negligible amplitude error and is suitable for highly integrated and broadband transceiver front-ends.

## Figures and Tables

**Table 1:** The impact of ECMML parameters on *C*.

	*W*_1_ + (*C_s_* +)	*W*_1_ − (*C_s_* −)	*d*_1_ + (*C_m_* −)	*d*_1_ − (*C_m_* +)	*n* +	*n* −
*C*	−	+	−	+	+	−

+ Value increases; − Value decreases.

**Table 2:** Performances comparison with other phase shifters.

Ref	Freq. (GHz)	RL (dB)	AI (dB)	Phase Shift (deg)	Techniques	Size ($\lambda_{g}^{2}$)
[20]	1.14–2.79	15	N/A	183 ± 2	parallel-coupled 3-line	0.0625
[26]	2.15–4.3	13.5	N/A	180 ± 4.5	parallel-coupled-line filter	0.192
[27]	1.6–2.4	7.5 *	<0.25	180 ± 4.2	edge-coupled delay line	0.258
[28]	3.12–3.96	15	N/A	180 ± 8	slotted substrate waveguide	1.8
[29]	1.45–3.28 **^#^**	10	N/A	180 ± 1.5	Slotlines-based Schiffman	0.5
[30]	8–11	10	N/A	180 ± 4.5	high/low pass	0.063
[31]	12–18	17.5	N/A	180 ± 6	high/low pass	0.11
This work ^$^	10–20	12	<0.1	179.7 ± 0.3	ECMML & ATL	0.0154

* Estimated value from figure; ^#^ Phase deviation bandwidth; ^$^ Simulation with measured transistors; AI for Amplitude difference.

## Data Availability

The data that support the findings of this study are available from the author upon reasonable request.
